# Growth factors with valproic acid restore injury-impaired hearing by promoting neuronal regeneration

**DOI:** 10.1172/jci.insight.139171

**Published:** 2021-11-22

**Authors:** Takahiro Wakizono, Hideyuki Nakashima, Tetsuro Yasui, Teppei Noda, Kei Aoyagi, Kanako Okada, Yasuhiro Yamada, Takashi Nakagawa, Kinichi Nakashima

**Affiliations:** 1 Department of Stem Cell Biology and Medicine and; 2 Department of Otorhinolaryngology, Graduate School of Medical Sciences, Kyushu University, Higashi-ku, Fukuoka, Japan.; 3 Division of Stem Cell Pathology, Center for Experimental Medicine and Systems Biology, Institute of Medical Science, University of Tokyo, Minato-ku, Tokyo, Japan.

**Keywords:** Neuroscience, Stem cells, Adult stem cells, Neuronal stem cells

## Abstract

Spiral ganglion neurons (SGNs) are primary auditory neurons in the spiral ganglion that transmit sound information from the inner ear to the brain and play an important role in hearing. Impairment of SGNs causes sensorineural hearing loss (SNHL), and it has been thought until now that SGNs cannot be regenerated once lost. Furthermore, no fundamental therapeutic strategy for SNHL has been established other than inserting devices such as hearing aids and cochlear implants. Here we show that the mouse spiral ganglion contains cells that are able to proliferate and indeed differentiate into neurons in response to injury. We suggest that SRY-box transcription factor 2/SRY-box transcription factor 10–double-positive (Sox2/Sox10–double-positive) Schwann cells sequentially started to proliferate, lost Sox10 expression, and became neurons, although the number of new neurons generated spontaneously was very small. To increase the abundance of new neurons, we treated mice with 2 growth factors in combination with valproic acid, which is known to promote neuronal differentiation and survival. This treatment resulted in a dramatic increase in the number of SGNs, accompanied by a partial recovery of the hearing loss induced by injury. Taken together, our findings offer a step toward developing strategies for treatment of SNHL.

## Graphical abstract

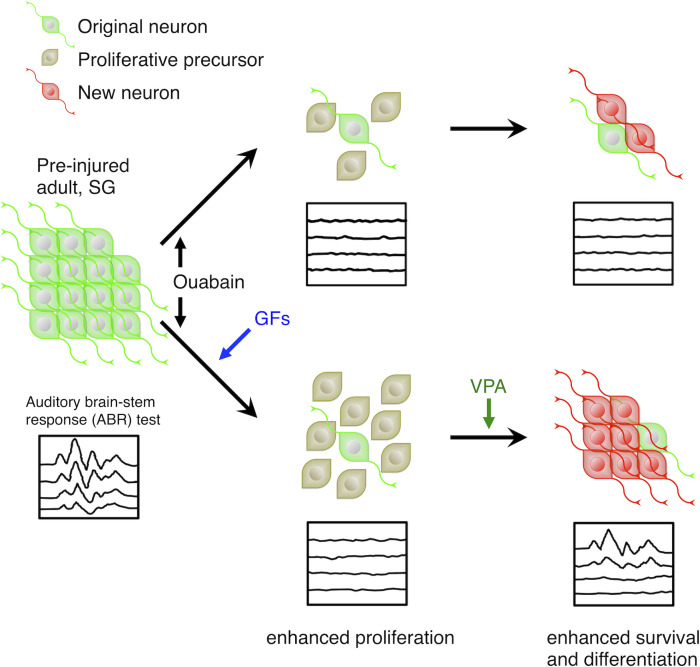

## Introduction

Spiral ganglion (SG) neurons (SGNs) are the primary auditory neurons and play a critical role in transmitting auditory information from sensory hair cells to the brain (1). Degeneration of SGNs is associated with aging, genetic mutation, and cochlear injuries caused by noise or ototoxic drug exposure and results in permanent sensorineural hearing loss (SNHL) (2, 3). In adult mammals, repair of damaged SGNs is very limited (4), but if SGNs could be replaced or regenerated, this would pave the way to restoring hearing in SNHL (5).

SGNs are predominantly bipolar neurons, and their development in mice begins around embryonic day (E) 9 in the otocyst (6). Neural stem/precursor cells (NS/PCs) differentiate and become postmitotic in the cochlear duct around E12, migrate a short distance from the otocyst to the SG by E14, send fibers to the organ of Corti, and eventually reach the inner/outer hair cells around E16 (7, 8). On the other hand, Schwann cells, which are peripheral glial cells, are derived from neural crest cells (NCCs) that arise in the dorsal neural tube around E9 (9). Some NCCs begin to migrate through immature connective tissue around E10–E11 and arrive at the SG through the modiolus (8), after which they become Schwann cell precursors around E14–E15 and then differentiate to immature Schwann cells around E18–E19 (10). Expression of the well-known Schwann cell marker SRY-box transcription factor 10 (Sox10), which is essential for the generation of the glial lineage from trunk crest cells, begins around E9–E12 (11). Previous work has revealed that these NCC-derived Schwann cells have a different origin from otocyst-derived SGNs (12). In mice, functional hearing begins to occur at postnatal day (P) 11, and the cochleae acquire adult-type morphology with terminally differentiated and quiescent cells by P20 (13). Recently, it has been reported that Schwann cells even in the adult mouse SG can proliferate in response to auditory system injury; however these proliferative cells do not differentiate into neurons, and there is thus no improvement in hearing (14).

Strategies for restoring lost hearing may include both transplanting exogenous cells and controlling endogenous cells to generate the requisite cells. Of the two, leverage of endogenous cells is preferable because it entails fewer concerns such as immune rejection (4, 15, 16). Therefore, despite the abovementioned negative report (14), we revisited and scrutinized a mouse model of inner ear injury and found that a very small number of proliferating cells did differentiate intrinsically into neurons in the adult mouse SG. However, even when we supplied growth factors (GFs) to enhance the proliferation of the cells, the eventual number of newly generated neurons was no higher than that without GFs.

Valproic acid (VPA; 2-propylpentanoic acid) is an established drug in the long-term treatment of epilepsy (17). Several studies have revealed that VPA can directly inhibit histone deacetylase (HDAC) activity and induce hyperacetylation of histones (18–20). We have previously shown that VPA drives NS/PCs into the neuronal lineage in preference to the glial lineage by a process involving HDAC inhibition (21, 22). Furthermore, it has also been shown that VPA has a neuroprotective function (23, 24). Taking these properties of VPA into account, we treated inner ear injury model mice with a combination of GFs and VPA and observed a dramatic increase in the resultant number of new neurons in the SG compared with GF treatment alone. Above all, the combinatorial treatment improved hearing function in the injured mice.

## Results

### Appearance of new SGNs after injury of the SG.

**Figure 1:**
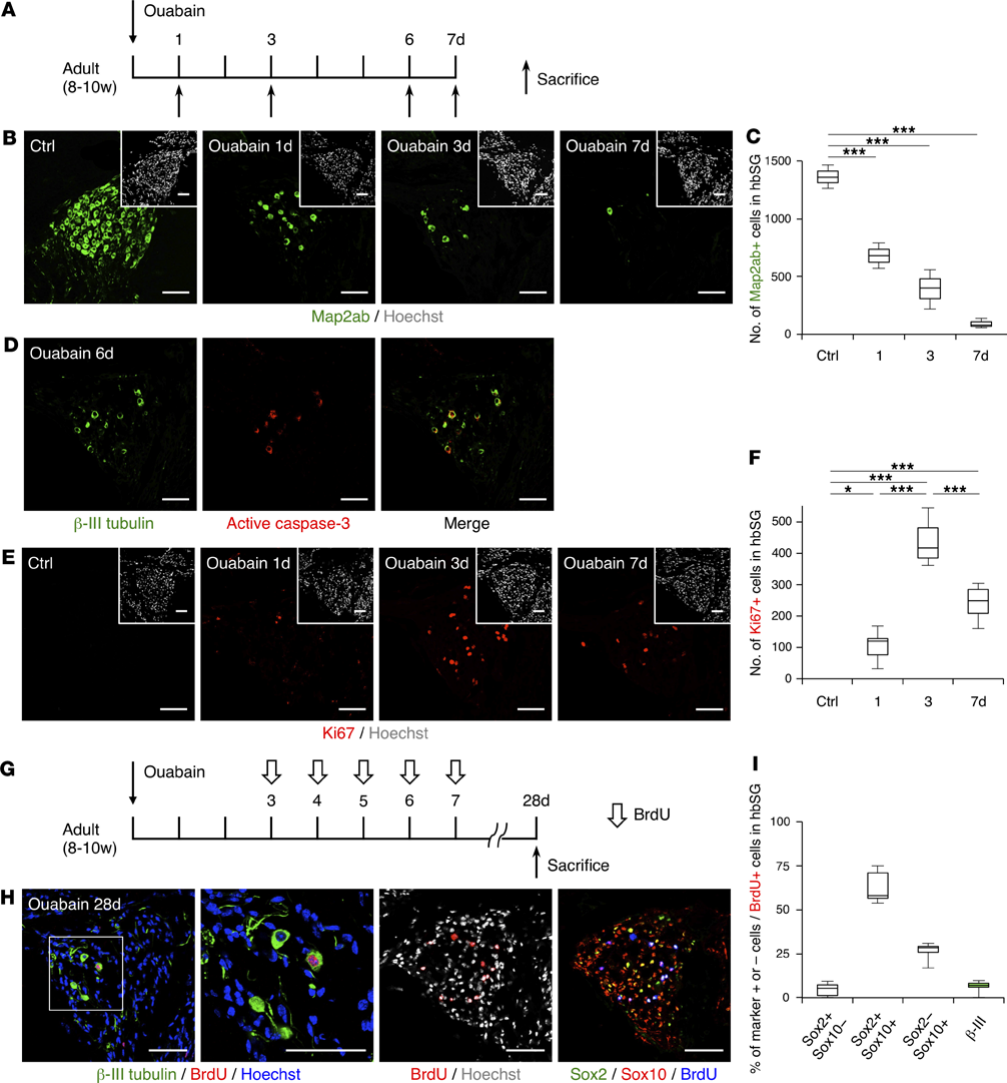
Emergence of new SGNs after SG injury. (**A**) Experimental scheme for investigating death of SGNs and proliferation of cells in response to injury. (**B**) Representative images of Map2ab-positive, mature SGNs after ouabain administration. Insets show H33258 nuclear staining of each field. (Scale bars, 50 μm.) (**C**) Quantification of the number of Map2ab-positive SGNs per hbSG, from data in **B** (*n* ≥ 3 animals each; error bars are mean ± SEM; ***P ≤ 0.001; 1-way ANOVA and Tukey’s multiple-comparison test). (**D**) Representative images of active cleaved caspase-3–positive SGNs on day 6 after ouabain administration. (Scale bars, 50 μm.) (**E**) Representative images of Ki67-positive proliferating cells that appeared after ouabain administration. Insets show H33258 nuclear staining of each field. (Scale bars, 50 μm.) (**F**) Quantification of the number of Ki67-positive cells per hbSG, from data in **E** (*n* ≥ 3 animals each; error bars are mean ± SEM; *P ≤ 0.05, ***P ≤ 0.001; 1-way ANOVA and Tukey’s multiple-comparison test). (**G**) Experimental scheme for investigating the fate on day 28 of cells that had been labeled with BrdU during days 3–7 after SG injury. (**H**) The 2 left images are representative of β-III tubulin/BrdU–double-positive newborn neurons. The area outlined by a white square is enlarged in the right panel. The 2 right images are representative of proliferating Sox2- and/or Sox10-positive Schwann cells. (Scale bars, 50 μm.) (**I**) Quantification of the indicated marker-positive/negative cells, from data in **H** (*n* ≥ 3 animals; error bars are mean ± SEM).

Although the adult mouse SG is mostly composed of quiescent SGNs and Schwann cells, both of which are nonproliferative under physiological conditions (6, 12, 25), it has been reported that Schwann cells can proliferate after injury (14, 26). Therefore, we first sought to confirm that quiescent cells in the SG resume proliferation in response to injury. To this end, we administered ouabain, a Na^+^/K^+^-ATPase inhibitor that can selectively damage SGNs (26–28), to the left inner ear of adult (8–10 weeks old) mice through the round window (14) and used their right ear as an intact control. Because the SG forms a seamless, spiral, tubelike structure in the cochlea, precluding accurate quantification of the total cell number in the SG of a cochlea, we counted the number of cells only in the half of the basal SG (hbSG) on the side opposite to the round window for quantification in this study (see Methods). When we examined the mice on days 1, 3, and 7 after ouabain administration, we observed a substantial decrease in the number of mature neuron marker Map2ab-positive cells in the ouabain-treated SG (Figure 1, A–C). Ouabain is known to induce apoptosis of SGNs (28), and we confirmed that active cleaved caspase-3–positive cells were abundant in the residual SGN population on day 6 after ouabain administration (Figure 1D). We also found that cells positive for Ki67, a marker for proliferating cells, despite not existing in the intact SG, indeed appeared and that their number was highest on day 3 after ouabain treatment (Figure 1, E and F). SGNs and Schwann cells are known to have developmentally different origins (12), and Sox2, an NS/PC marker, is expressed in the otocyst and downregulated after birth (5). A previous study suggested that Sox2, as well as Sox10, can be used as a Schwann cell marker in the adult mouse SG and that injury-associated proliferating cells become Schwann cells but not SGNs (14). To confirm that these proliferating cells actually differentiated only into Schwann cells, we injected BrdU into adult mice once a day from days 3 to 7 to label proliferating cells and sacrificed mice on day 28 after ouabain treatment (Figure 1G). Although the majority of BrdU-labeled cells on day 28 were indeed Sox2/Sox10–double-positive or Sox10–single-positive Schwann cells, we were surprised to also detect a small number of β-III tubulin/BrdU–double-positive cells (Figure 1, H and I). These results indicate that a few of the cells that were proliferating on days 3 to 7 after ouabain administration differentiated into neurons by day 28 and that cells in the adult SG have the potential to regenerate SGNs in response to injury.

### Analysis of the origin of new SGNs after injury.

**Figure 2:**
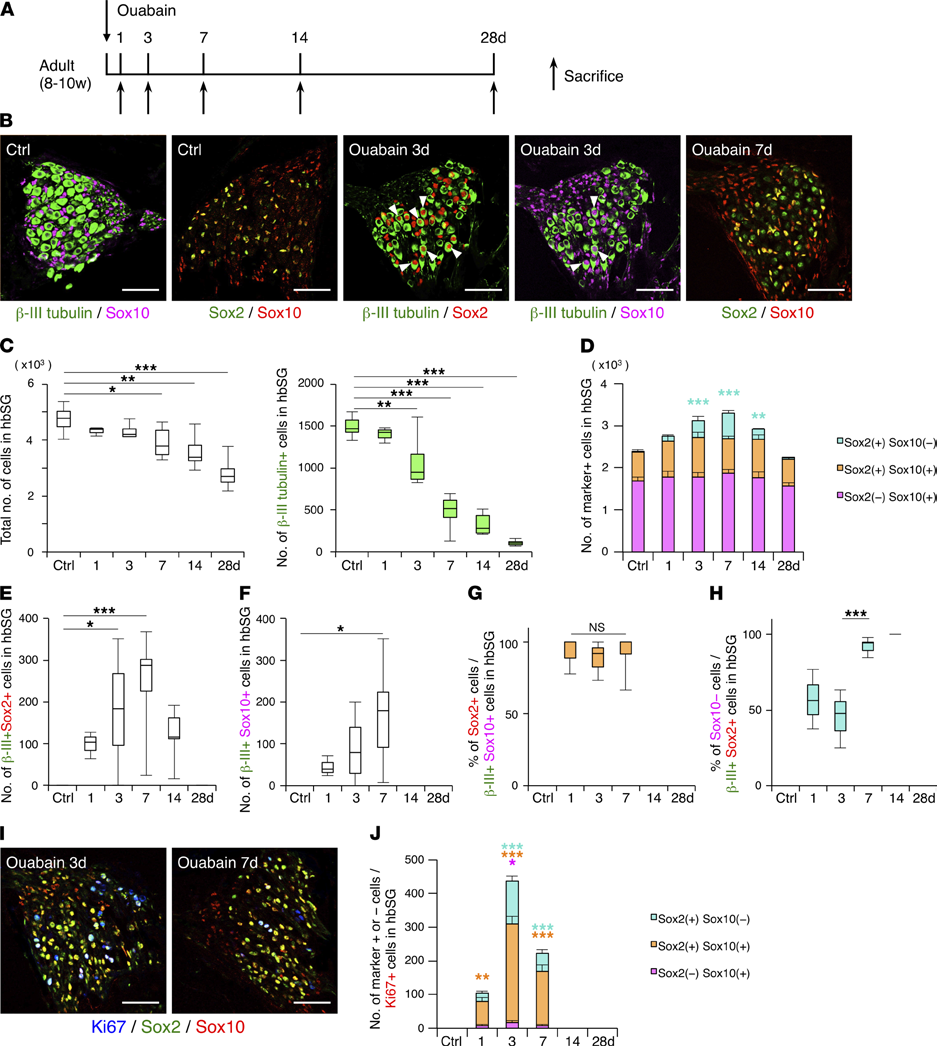
Analyzing origin of newly generated SGNs. (**A**) Timeline for investigating the origin of new SGNs. (**B**) Marker^+^ cells in the SG of Ctrl and on days 3 and 7. On day 3, β-III tubulin^+^Sox2^+^ cells and β-III tubulin^+^Sox10^+^ cells were found (arrowheads). (Scale bars, 50 μm.) (**C**) Total cell and β-III tubulin^+^ neuron number per hbSG of Ctrl and on days 1 to 28. (**D**) Sox2^+^Sox10^−^, Sox2^+^Sox10^+^, and Sox2^−^Sox10^+^ cell numbers per hbSG of Ctrl and on days 1 to 28. (**E** and **F**) β-III tubulin^+^Sox2^+^ and β-III tubulin^+^Sox10^+^ cell number per hbSG of Ctrl and on days 1 to 28. (**C**–**F**) *n* ≥ 3 animals each; error bars are mean ± SEM; *P ≤ 0.05, **P ≤ 0.01, ***P ≤ 0.001; 1-way ANOVA and Dunnett’s multiple-comparison test compared with the control. (**G**) Sox2^+^ cell proportion among β-III tubulin^+^Sox10^+^ cells per hbSG of Ctrl and on days 1 to 28 (*n* ≥ 3 animals each; error bars are mean ± SEM; 1-way ANOVA and Tukey’s multiple-comparison test). (**H**) Sox10^−^ cell proportion among β-III tubulin^+^Sox2^+^ cells per hbSG of Ctrl and on days 1 to 28 (*n* ≥ 3 animals each; error bars are mean ± SEM; ***P ≤ 0.001; 1-way ANOVA and Tukey’s multiple-comparison test). (**I**) Ki67^+^, Sox2^+^, and Sox10^+^ cells in the SG on days 3 and 7. (Scale bars, 50 μm.) (**J**) Sox2^+^Sox10^−^, Sox2^+^Sox10^+^, and Sox2^−^Sox10^+^ cell number in Ki67^+^ cells per hbSG of Ctrl and on days 1 to 28 (*n* ≥ 3 animals each; error bars are mean ± SEM; *P ≤ 0.05, **P ≤ 0.01, ***P ≤ 0.001; 1-way ANOVA and Tukey’s multiple-comparison test compared with the control).

To determine which cells are the source of these newly generated SGNs, we administered ouabain to the left inner ear of adult mice and sacrificed them on days 1, 3, 7, 14, and 28 after the injury (Figure 2A). In the intact hbSG in the right ear, there were about 5000 cells, of which about 1500 were β-III tubulin–positive SGNs and about 2400 were Sox10-positive Schwann cells (Figure 2, C and D). About 750 Sox2-positive cells existed in the intact hbSG, and almost all of these were Sox10 positive, making up 30% of the total Sox10-positive Schwann cells (Figure 2, B and D). In addition, β-III tubulin–positive SGNs and Sox10-positive Schwann cells were mutually exclusive in the control (Figure 2B). After ouabain administration, the total number of cells in the hbSG gradually decreased by day 28, and we found that the number of lost cells approximately corresponded to the number of decreased β-III tubulin–positive cells (Figure 2C). Interestingly, in the injured SG, we identified β-III tubulin/Sox2–double-positive and β-III tubulin/Sox10–double-positive cells, implying that these cells were in a state of transition into neurons (Figure 2, B, E, and F). Furthermore, almost all Sox2-positive cells were also positive for Sox10 in the intact SG, while Sox2–single-positive cells appeared and reached their highest number on day 7 after injury (Figure 2, B and D). On the other hand, the numbers of Sox2/Sox10–double-positive and Sox10–single-positive cells did not change significantly after injury (Figure 2D). Importantly, most β-III tubulin/Sox10–double-positive cells were also Sox2 positive (Figure 2, F and G), but almost all β-III tubulin/Sox2–double-positive cells on days 7 and 14 after injury were Sox10 negative (Figure 2, E and H). We further found that among Ki67-positive proliferating cells in the injured SG, Sox2–single-positive and Sox2/Sox10–double-positive cells significantly increased, with their highest numbers on day 3 after injury (Figure 2, I and J). Overall, taking into account that postmitotic neurons do not divide and that Sox10–single-positive Schwann cells rarely proliferate in response to ouabain treatment (Figure 2J), our interpretation of these observations is that after injury, Sox2/Sox10–double-positive Schwann cells sequentially started to proliferate, expressed β-III tubulin, lost Sox10 expression and stopped proliferating by day 14, and eventually became Sox2-negative and β-III tubulin–single-positive SGNs.

### Strategy to enhance SGN regeneration and achieve hearing recovery.

A previous study showed that hearing ability was maintained if at least ~30% of the original SGNs survived during aging and/or after insults (29). In our SG injury model, however, less than 10% of the original number of SGNs remained on day 28 after ouabain administration (Figure 2C, ~100 cells out of ~1500 cells), leading to severe hearing loss (see below). Moreover, even though we found that new neurons were generated after SG injury, the eventual total of about 100 neurons in hbSG, including those newly generated, was evidently too low for the SG to attain functional recovery. We therefore next attempted to increase the number of new neurons after injury to see if we could improve the lost hearing ability of injured mice.

**Figure 3:**
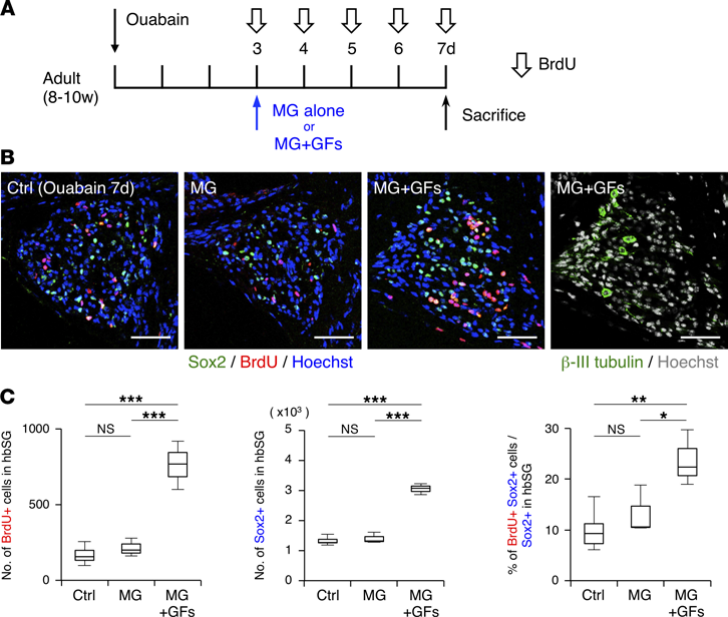
GFs enhance the proliferation of new SGN precursors after injury. (**A**) Experimental scheme to examine the effect of GFs on proliferation of cells in the injured SG. BrdU was injected intraperitoneally at 50 mg/kg every day on days 3 to 7. Ctrl is SG of mice without reoperation and sacrificed on day 7 after ouabain administration. To examine the effect of Matrigel (MG), we compared MG alone and MG+GFs; in both treatments mice were reoperated on day 3 and sacrificed on day 7. (**B**) Representative images of the indicated marker-positive cells in Ctrl, MG alone–, and MG+GF–treated SGs on day 7 after ouabain administration. (Scale bars, 50 μm.) (**C**) Quantification of the number of BrdU- and Sox2-positive cells, and the proportion of BrdU/Sox2–double-positive cells among Sox2-positive cells, per hbSG of Ctrl, MG alone, and MG+GF mice on day 7 after ouabain administration (*n* = 3 animals each; error bars are mean ± SEM; *P ≤ 0.05, **P ≤ 0.01, ***P ≤ 0.001; 1-way ANOVA and Tukey’s multiple-comparison test).

Since it is generally accepted that terminally differentiated neurons do not proliferate, we tried to enhance the proliferative activity of precursors of the new SGNs after injury by administering GFs. Because epidermal growth factor (EGF) and basic fibroblast growth factor (bFGF) are known as the 2 most potent growth factors for NS/PCs (30–32), we administered ouabain to the inner ear of adult mice, reoperating on day 3 after injury (when proliferating cells are most abundant after injury; Figure 1F and Figure 2J) to administer GFs (EGF + bFGF with Matrigel [MG]) via the round window (Figure 3A). We also injected BrdU daily from days 3 to 7 after injury to label proliferating cells in the SG, then sacrificed the mice 2 hours after the last BrdU injection on day 7 (Figure 3A). In this experimental setting, we observed greatly increased numbers of BrdU- and Sox2-positive cells (Figure 3, B and C), suggesting that treatment with GFs indeed increased proliferating precursors in the hbSG after injury.

**Figure 4:**
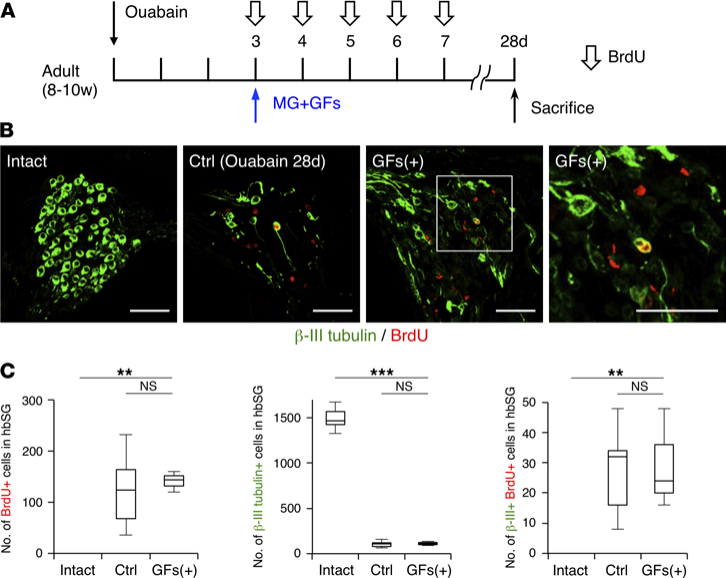
Effect of treatment with GFs alone. (**A**) Experimental scheme for investigating the fate of BrdU-positive cells with GF treatment on day 28 after ouabain administration. BrdU was injected intraperitoneally at 50 mg/kg every day on days 3 to 7. Intact mice did not receive any operation or reagent treatment. Ctrl is mice without reoperation and sacrificed on day 28 after ouabain administration. (**B**) Representative images of the indicated marker-positive cells in SGs of intact, Ctrl, and MG+GF–treated mice on day 28 after ouabain administration. The area outlined by a white square is enlarged in the rightmost panel. (Scale bars, 50 μm.) (**C**) Quantification of the number of BrdU-positive cells, β-III tubulin–positive neurons, and β-III tubulin/BrdU–double-positive new neurons per hbSG of intact, Ctrl, and MG+GF mice (*n* = 3 animals each; error bars are mean ± SEM; **P ≤ 0.01, ***P ≤ 0.001; 1-way ANOVA and Tukey’s multiple-comparison test).

To check whether these increased proliferating cells differentiated into neurons, we sacrificed mice on day 28 after injury (Figure 4A). Contrary to our expectations, the numbers of BrdU-positive cells, newly generated BrdU/β-III tubulin–double-positive cells, and even total β-III tubulin–positive SGNs were no higher in the GF-treated hbSG than in the untreated hbSG (Figure 4, B and C).

**Figure 5:**
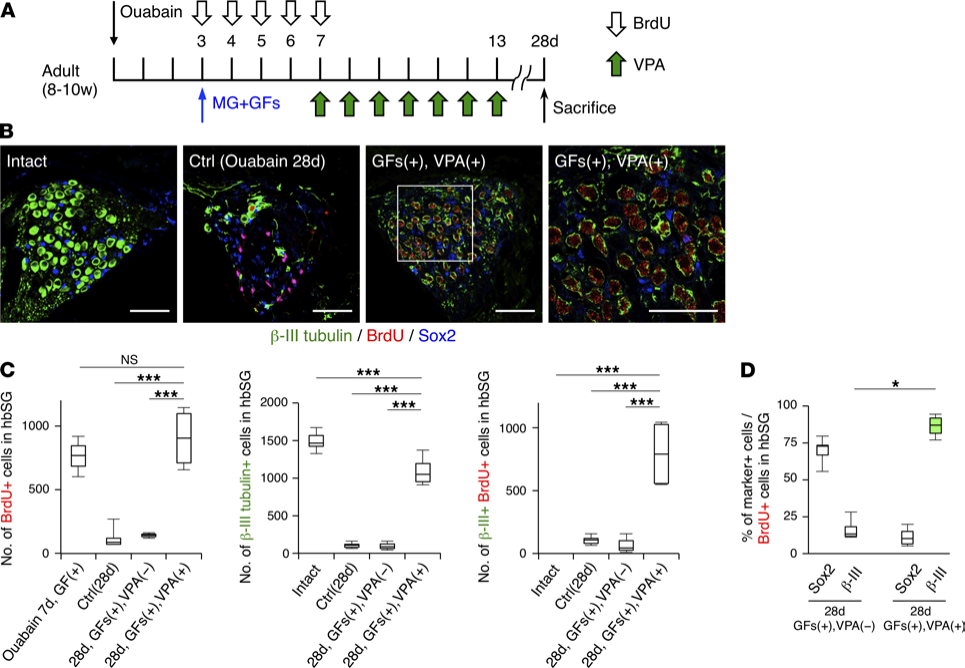
VPA promotes SGN regeneration. (**A**) Experimental scheme to investigate neuronal differentiation of proliferating cells in response to combined treatment with GFs and VPA. BrdU was injected intraperitoneally at 50 mg/kg every day on days 3 to 7. Intact mice did not receive any operation or treatment with reagents. Ctrl is mice without reoperation and sacrificed on day 28 after ouabain administration. (**B**) Representative staining of cells in intact, Ctrl, and GF+VPA–treated SGs on day 28 after ouabain administration. The area outlined by a white square is enlarged to the right. (Scale bars, 50 μm.) (**C**) Quantification of the number of BrdU-positive, β-III tubulin–positive, and β-III tubulin/BrdU–double-positive new neurons per hbSG of intact, Ctrl, GF alone–, and GF+VPA–treated mice. In the leftmost graph, the number of BrdU-positive cells in the hbSG of mice administered with GFs and sacrificed on day 7 is also indicated for comparison (*n* = 3 animals each; error bars are mean ± SEM; ***P ≤ 0.001; 1-way ANOVA and Tukey’s multiple-comparison test). (**D**) Quantification of the percentage of Sox2-positive and β-III tubulin–positive cells among BrdU-positive cells in the hbSG of mice treated with GFs with or without VPA after ouabain injury (*n* = 3 animals each; error bars are mean ± SEM; *P ≤ 0.05; 2-tailed Student’s *t* test).

Having shown that treatment with GFs alone, which could enhance precursor proliferation, was insufficient to facilitate eventual regeneration of SGNs, we then treated the injured mice with a combination of GFs and VPA. VPA, an HDAC inhibitor that is used as an antiepileptic drug, is known to induce neuronal differentiation of proliferating cells and to promote neuronal survival (21–24, 33). To examine whether VPA improves neuronal differentiation and survival in the ouabain-damaged SG, we treated the injured mice with GFs on day 3 and injected VPA intraperitoneally from day 7, when proliferating cells were shown to have increased significantly (Figure 3C), for 1 week, then sacrificed them on day 28 after injury (Figure 5A). Surprisingly, the combined treatment with GFs and VPA dramatically increased the ratio of β-III tubulin–positive cells among BrdU-positive cells and the number of BrdU/β-III tubulin–double-positive cells in the injured hbSG (Figure 5, B–D), indicating that VPA promoted both neuronal differentiation and survival of proliferating cells and/or new SGNs. In support of these results, we detected active cleaved caspase-3/β-III tubulin–double-positive cells in mice with GF without VPA treatment 10 days after ouabain injury, whereas the number of these cells was greatly reduced in those with VPA treatment, suggesting that VPA enhances neuronal survival (Supplemental Figure 1; supplemental material available online with this article; https://doi.org/10.1172/jci.insight.139171DS1). More importantly, the total number of β-III tubulin–positive SGNs in the hbSG generated with the combinatorial treatment on day 28 after injury recovered to almost 75% of the number in the intact hbSG (Figure 5C).

**Figure 6:**
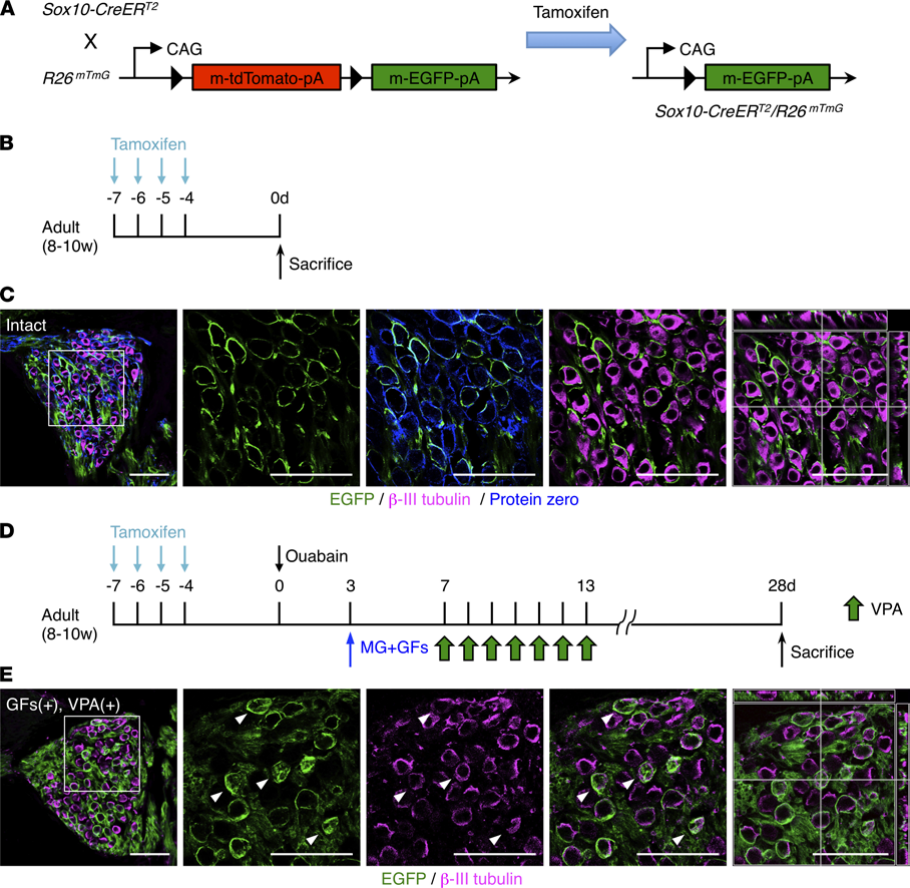
Identification of the source cells of new SGNs. (**A**) Schematic representation of tamoxifen-inducible EGFP expression in Sox10-CreER^T2^ R26^mTmG^ transgenic mice. mT, membrane-targeted tandem dimer Tomato; mG, membrane-targeted enhanced green fluorescent protein; pA, polyadenylation sequence. (**B**) Experimental scheme for assessing EGFP expression in Schwann cells in intact mice. Adult Sox10-CreER^T2^ R26^mTmG^ mice were administered with tamoxifen daily for 4 days and sacrificed 4 days after the last tamoxifen administration. (**C**) Representative images of EGFP-positive Schwann cells after tamoxifen administration. The area outlined by a white square is enlarged in the panels to the right. The rightmost image is an ortho-view of *Z*-stack images. (Scale bars, 50 μm.) (**D**) Experimental scheme for lineage-tracing analysis of the new SGNs in response to combined treatment with GFs and VPA. (**E**) Representative images of new SGNs transformed from EGFP-positive Schwann cells by GFs and VPA treatment. The area outlined by a white square is enlarged in the panels to the right. EGFP/β-III tubulin–double-positive cells were found (arrowheads). The rightmost image is an ortho-view of *Z*-stack images. (Scale bars, 50 μm.)

To precisely determine the original cell source of the newly generated SGNs, after injury, with GF and VPA treatment, we next performed lineage-tracing analysis using Sox10-CreER^T2^ mice (34) crossed with mTmG mice (35) (Sox10-CreER^T2^ R26^mTmG^). We first induced EGFP expression in Sox10-expressing cells by injecting tamoxifen on 4 consecutive days into the intact Sox10-CreER^T2^ R26^mTmG^ mice and sacrificed the mice 4 days after the last injection (Figure 6, A and B). We showed that all EGFP-positive cells were also positive for the Schwann cell marker Protein zero in the SG of these mice (Figure 6C). The EGFP-positive cells surrounded β-III tubulin–positive cells, but none of these cells were positive for β-III tubulin in the SG of intact mice (Figure 6C). By contrast, in tamoxifen-pretreated, ouabain-injured mice with the combinatorial treatment of GFs and VPA, we found EGFP/β-III tubulin–double-positive SGNs at 28 days after injury (Figure 6, D and E). These results indicate that the newly generated SGNs with the combinatorial treatment after injury originated from Sox10- and Protein zero–positive Schwann cells.

**Figure 7:**
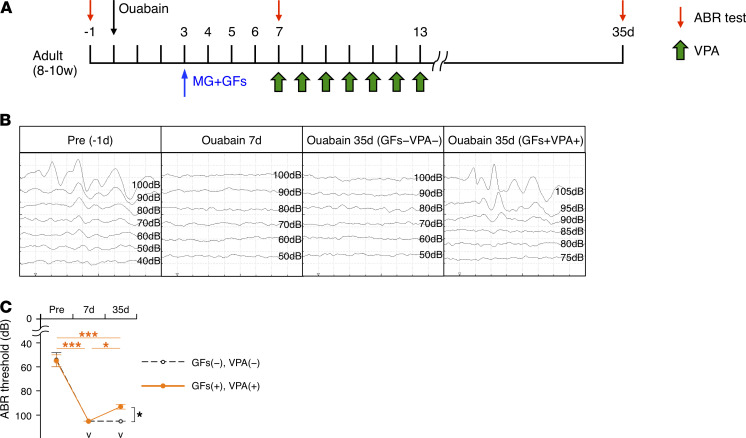
Enhanced SGN regeneration leads to hearing recovery. (**A**) Experimental scheme for evaluating functional recovery of hearing by the ABR test. ABRs to clicks were recorded before ouabain exposure (Pre) and on days 7 and 35 after ouabain exposure in mice with or without the combined GFs and VPA treatment. (**B**) Prior to ouabain treatment (Pre), ABR wave thresholds could be recorded, but by day 7 after ouabain exposure, the ABR was completely absent. Without treatment, the ABR remained absent 35 days after the injury, whereas it was restored by combinatorial treatment with GFs and VPA. (**C**) Quantification of ABR test results. Orange circles are the results of ABR tests in mice treated with combined GFs and VPA after ouabain exposure. Open circles are the results of ABR tests in mice that were not treated with GF or VPA after ouabain exposure. The v marks indicate scale-out, complete deafness (*n* ≥ 3 animals each; error bars are mean ± SEM; *P ≤ 0.05, ***P ≤ 0.001; 1-way ANOVA and Tukey’s multiple-comparison test).

Having succeeded in obtaining a large number of regenerated neurons in the injured SG, we finally evaluated functional recovery of hearing using the auditory brainstem response (ABR) test (Figure 7A). In all healthy mice before ouabain administration, ABR wave thresholds were observed (~60 dB), showing the ABR (Figure 7, B and C). The ABR was completely absent in mice 7 days after ouabain exposure with or without GF treatment (Figure 7, B and C). However, in mice 35 days after ouabain exposure, those without combined GF and VPA treatment remained unresponsive in the ABR test, while those treated with GFs and VPA reacquired responsiveness (Figure 7, B and C), indicating that the treatment with GFs and VPA resulted in a substantial, albeit not complete, improvement in hearing compared with nontreatment (~90 dB).

## Discussion

Despite much research (6, 8, 36), a fundamental treatment for replacing or regenerating SGNs for SNHL has not yet been established. For example, Lang et al. showed that proliferative Schwann cells appear even in the adult SG after injury, but these proliferating cells did not differentiate into neurons, the most important cell type for improving hearing function (14). However, in the present study, we found that a small number of new SGNs appeared after inner ear injury using ouabain when we analyzed the damaged SG in detail. We also proposed that some Sox2/Sox10–double-positive cells began to proliferate and subsequently lost Sox10 expression, then differentiated into SGNs, but the number of newly generated neurons was apparently too small to restore lost hearing because at least ~30% of the original SGNs must survive to maintain hearing ability during aging or after insults (29). Therefore, we first attempted to enhance proliferation of cells using GFs, but this treatment failed to increase the final number of neurons. However, we eventually attained a dramatic increase in the number of newly generated SGNs by means of a combined treatment of the injured mice with GFs and VPA, a reagent that is known to promote neuronal differentiation and survival (21–23, 33). VPA is a well-known HDAC inhibitor (18–20), and we showed that VPA treatment indeed increased histone H3 acetylation in the SG of mice (Supplemental Figure 2). We have previously shown that VPA induces the expression of the proneural transcription factor NeuroD1 in neural stem cells to promote neural differentiation (21). Therefore, taking into account a study showing that NeuroD1 is required for the survival of new neurons (24), it is conceivable that VPA-induced NeuroD1 plays critical roles in the promotion of neuronal differentiation and survival of cells in the SG after injury, although further investigation is needed to elucidate in detail the molecular mechanism of the VPA-induced effective regeneration of SGNs.

Regarding neuronal subtypes in the SG, Prox1-positive type I SGNs represent 90%–95% of the population and project to the inner hair cells, which encode acoustic information by transducing mechanical stimuli into electrochemical signals (37). On the other hand, Peripherin-positive type II SGNs, making up the remaining 5%–10% of the population, project to the outer hair cells, which promote sound amplification and frequency selectivity (37). Therefore, regeneration of type I SGNs is considered more important than that of type II SGNs for direct auditory function. In this context, it has been shown that Peripherin-positive type II SGNs are not affected by ouabain treatment and are maintained even after injury (28). We showed that the small number of type II SGNs was maintained after ouabain injury and that almost all new neurons generated by our combined treatment were Peripherin-negative and Prox1-positive type I SGNs (Supplemental Figure 3). The improved hearing attained by our treatment is thus probably attributable to the regeneration of type I SGNs.

Although we could improve impaired hearing function in injured mice with our combined treatment, their recovery was incomplete. Possible reasons for this include 1) 35 days after injury may be too soon to observe complete functional recovery even with the combined treatment if maturation of new SGNs requires more time, and 2) projection of new SGNs to hair cells may have been insufficient for complete recovery, in which case recovery may be enhanced by further treatment with neurite outgrowth–promoting factors such as brain-derived neurotrophic factor and neurotrophin-3 (32, 38). These considerations warrant future investigation with the goal of achieving complete functional restoration of hearing after injury. Meanwhile, since we have shown that the SG has intrinsic potential to regenerate lost SGNs, identified an effective treatment to enhance their regeneration, and demonstrated that the treatment ameliorated hearing in injured mice, we believe that our findings provide a new avenue for the development of therapeutic strategies to counter SNHL caused by SGN degeneration.

A recent study showed that overexpression of Lin28 in proteolipid protein 1–positive glial cells of the SG reprograms the cells into neurons following ouabain injury, although neurons barely increase without injury (39). As we have shown, without injury by ouabain, no new neurons were generated, but the addition of GFs and VPA after injury increased the number of neurons and improved hearing function. Taken together, our present findings and this new report (39) indicate that by intervening after injury, it is possible to generate new neurons.

## Methods

### Animals.

ICR male mice (Japan SLC) were used for all, except for Figure 6’s, experiments. We used male mice of the Sox10-CreER^T2^ R26^mTmG^ transgenic line for Figure 6, established by crossing Sox10-CreER^T2^ (34) with ROSA^mTmG^ mice (35) (Jackson Laboratory stock 007676). Prior to Cre recombination, fluorescence from mT expression is widespread in cells/tissues of the Sox10-CreER^T2^ R26^mTmG^ transgenic mice. However, cells expressing the Sox10 promoter–driven and tamoxifen-activated Cre recombinase (and future cell lineages derived from these cells) display mG fluorescence replacing the red fluorescence (Figure 6A). All efforts were made to minimize animal suffering and to reduce the number of animals used. Animals were housed under a 12-hour light/12-hour dark cycle and fed ad libitum. All aspects of animal care and treatment were carried out according to the guidelines of the Experimental Animal Care Committee of Kyushu University.

### Ouabain exposure.

Surgical procedures for administering ouabain via the round window followed previous methods (14, 26). Briefly, 8- to 10-week-old mice were anesthetized with a mixture of 4 mg/kg midazolam, 0.3 mg/kg medetomidine, and 5 mg/kg butorphanol. A postauricular incision was made, part of the salivary gland was resected, and the bone outside the middle ear bulla was exposed. Part of this bone was broken to reveal the round window niche inside the bulla. A filter paper impregnated with 50 μL of 3 mM ouabain (TOCRIS, diluted with normal saline) was placed on the round window membrane and left for 10 minutes. The total time of ouabain exposure was 60 minutes; the ouabain solution was wiped away with filter paper wicks every 10 minutes and replaced with fresh solution. The broken middle ear bulla surface was then covered with the resected salivary gland, and the incision was closed to complete the operation. On the first day after ouabain administration, the mice rotated severely, but they stopped rotating by around the third day. Ouabain-treated mice that did not rotate severely were considered inadequately exposed to the drug and were excluded from subsequent experimentation. The left ear was operated on while the right ear served as a control.

### Reoperation and administration of GFs.

On the third day after ouabain administration, the mice were anesthetized again, the postauricular incision was opened, and the overlying salivary gland was removed. The inside of the middle ear bulla was cleaned and the round window niche identified, after which 100 μL of GFs (EGF and bFGF, 20 ng/mL each; Peprotech), mixed with MG to prevent immediate efflux, was placed inside the bulla. The bulla surface was again covered with the salivary gland, and the procedure was completed by closing the incision. After reoperation, the severe rotation of the mice was not observed.

### VPA injection.

Some of the mice that had been administered GFs in reoperation on day 3 after ouabain treatment received an intraperitoneal injection of 300 mg/kg VPA (MilliporeSigma) once daily for 1 week, starting on day 7 after ouabain administration.

### Tamoxifen administration.

Tamoxifen (MilliporeSigma, T5648) was dissolved in sesame oil at 25 mg/mL. To induce EGFP expression in Sox10-expressing Schwann cells, 5 mg of tamoxifen was orally administered to 8- to 10-week-old Sox10-CreER^T2^ R26^mTmG^ mice with a feeding needle daily for 4 days.

### Cochlear sections and cell counts.

The mice were sacrificed, and their cochleae were removed, fixed in 4% paraformaldehyde (PFA) overnight at 4°C, cryoprotected in 10%–30% sucrose in PBS, and embedded in OCT compound. Mice older than P7 were anesthetized and transcardially perfused with PBS and 4% PFA before sacrifice. The cochleae of mice beyond P7 were decalcified with 0.5 M EDTA (pH 8.0) for 3 days at 4°C prior to sucrose cryoprotection. The embedded tissues were sectioned parallel to the modiolus at 10 μm thickness on a cryostat. When 1 cochlea was sectioned at 10 μm, 24 sections containing the basal SG could be identified. We mounted every eighth section on 1 slide, and all sections were thus placed on a total of 8 slides and evaluated by immunostaining. Since the cochlea has a spiral structure, 2 basal SG regions were observed simultaneously in a single section. We therefore counted the number of cells only in the half of the basal SG on the side opposite the round window (hbSG). The number of cells of each type in the 3 hbSGs on 1 slide was counted separately and summed, and this number was multiplied by 8 to give the total number of cells in each hbSG.

### Immunohistochemistry.

We performed immunohistochemistry as described previously (40). Briefly, the sections were blocked for 1 hour at room temperature with blocking solution (5% FBS and 0.3% Triton X-100) and incubated overnight at 4°C with primary antibodies. For BrdU staining, sections were incubated with 2N HCl for 20 minutes at 37°C before blocking. Ki67, Sox10, Prox1, Peripherin, and sometimes BrdU staining was performed after antigen retrieval using target retrieval solution (DAKO) for 3 hours at 80°C. The antibodies used were mouse anti–β-III tubulin (1:500, T8578; MilliporeSigma), rabbit anti–β-III tubulin (1:500, PRB-435P; Covance), mouse anti-Sox2 (1:250, MAB2018; R&D Systems), rabbit anti-Sox2 (1:250, AB5603; MilliporeSigma), goat anti-Sox2 (1:250, AF2018; R&D Systems), goat anti-Sox10 (1:250, sc-17342; Santa Cruz Biotechnology), mouse anti-Ki67 (1:500, 550609; BD Pharmingen), rat anti-BrdU (1:500, OBT0030; ABD), mouse anti-Map2 (2a+2b) (1:500, M1406; MilliporeSigma), rabbit anti–cleaved caspase-3 (1:500, 9661; Cell Signaling Technology), rabbit anti–Myelin Protein Zero (1:500, bs-0337R; Bioss), mouse anti–acetyl histone H3 (1:500, MABI0010; MCA), goat anti-Prox1 (1:250, AF2727; R&D Systems), and rabbit anti-Peripherin (1:500, AB1530; MilliporeSigma). Sections were incubated for 2 hours with corresponding secondary antibodies, CF-488A donkey anti–mouse IgG (H+L) highly cross-adsorbed (1:500, 20014; Biotium), CF-488A donkey anti–rabbit IgG (H+L) highly cross-adsorbed (1:500, 20015; Biotium), CF-555 donkey anti–rabbit IgG (H+L) highly cross-adsorbed (1:500, 20038; Biotium), CF-568 donkey anti–rat IgG (H+L) highly cross-adsorbed (1:500, 20092; Biotium), CF-647 donkey anti–goat IgG (H+L) highly cross-adsorbed (1:500, 20048; Biotium), and Alexa Fluor 405–preadsorbed donkey anti–rabbit IgG H&L (1:500, ab175649; Abcam). Nuclei were stained using Hoechst 33258 (Nacalai Tesque). Fluorescence images were obtained on a confocal laser microscope (LSM 800; Zeiss).

### ABR test.

ABR testing was conducted in a sound-attenuated shielded booth under anesthesia. An evoked potential system (Neuropack M1, MEB-9204) was used to measure the threshold of the ABR. ABR thresholds were recorded using 3 silver needle electrodes placed subdermally over the vertex (active) and the bilateral retroauricular region (ground and reference) of each mouse. For click-evoked ABRs, alternative click sounds were presented to evoke an ABR, and the intensity of the stimulus varied in 5 or 10 dB stepwise decrements from 105 dB. ABR thresholds were measured prior to ouabain exposure and on days 7 and 35 following ouabain administration with or without GF and VPA treatment. We selected mice with an ABR threshold of about 60 dB, which was observed most frequently, and used them for further experiments to exclude the effect of original differences in hearing ability among individual mice when we performed the ABR test for mice with or without treatments.

### Statistics.

All the data presented in the text are shown as means ± SEM, and we used GraphPad Prism 5 for statistical analysis. For all box-and-whisker plots, plots depict the minimum and maximum values (whiskers), the upper and lower quartiles, and the median. The length of the box represents the interquartile range. For all experiments, *n* represents the number of replicates, and at least 3 independent experiments were conducted. Two-tailed, unpaired Student’s *t* tests were used to determine statistical significance when comparing 2 groups, and 1-way ANOVA followed by a Tukey’s multiple-comparison test or a Dunnett’s multiple-comparison test were used when comparing more than 2 groups. A *P* ≤ 0.05 was considered statistically significant.

### Study approval.

Animal protocols were reviewed and approved by Kyushu University Institutional Review Board for Animal Experiments (A29-274 and A19-341).

## Author contributions

TW, HN, TY, and KN designed the study, and TW, HN, and KN wrote the manuscript. T Noda advised on how to remove and stain the inner ear. YY generated the transgenic mice. HN, KA, KO, and T Nakagawa helped with data acquisition. TW performed all experiments and data analysis. All authors discussed the data and commented on the manuscript. KN provided funding and supervised the project.

## Supplementary Material

Supplemental data

## Version 1 11/22/2021

Electronic publication
