# Supplementary material for: Growth factors with valproic acid restore injury-impaired hearing by promoting neuronal regeneration
Source: JCI Insight. 2021 Nov 22;6(22):e139171. doi: 10.1172/jci.insight.139171 (PMC8663787; doi:10.1172/jci.insight.139171)
Supplement: Supplemental data [file jciinsight-6-139171-s065.pdf]

Supplemental data

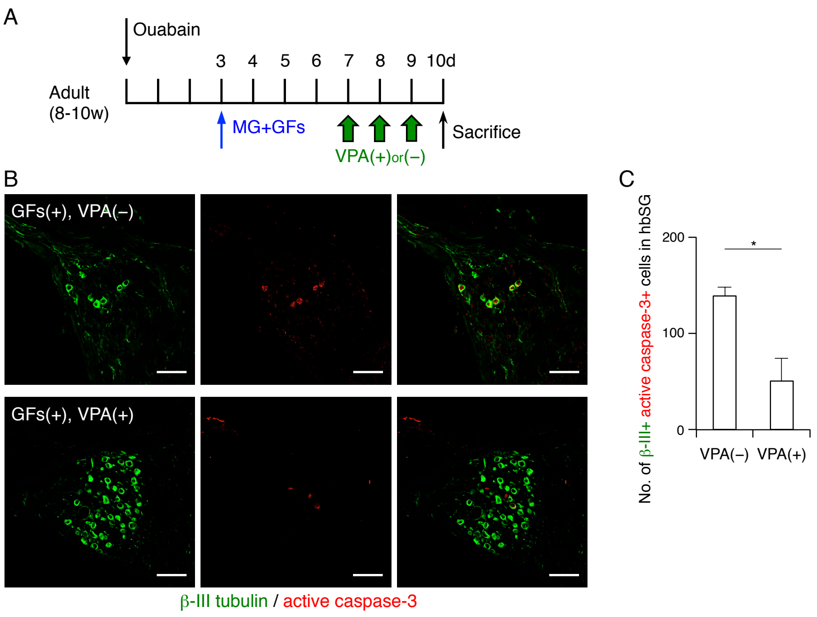

Supplemental Figure 1. VPA promotes survival of β-III tubulin-positive neurons. (**A**) Experimental scheme for investigating survival of SGNs in response to VPA administration. (**B**) Representative images of active caspase-3-positive SGNs, comparing with or without VPA treatment 10 days after ouabain administration. In both cases, GFs were administered three days after ouabain administration. (Scale bars, 50 μm.) (**C**) Quantification of the total number of active caspase-3-positive cells per hbSG, from data in (**B**) ($n \geq 3$ animals each; error bars are mean ±SEM; $^{*}P \leq 0.05$; Student's t test).

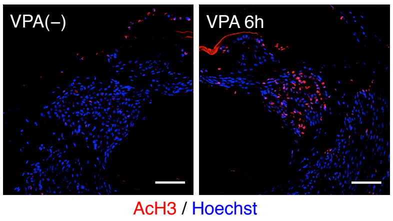

Supplemental Figure 2. VPA treatment increases histone acetylation in the SG. Left: Representative image of a VPA-untreated SG section stained with anti-acetylated histone H3 (AcH3) antibody (red). Nucli were conterstained with Hoechst (blue). Right: Representative image of a SG section 6 hours after VPA treatment, stained with anti-AcH3 antibody (red). Nucli were conterstained with Hoechst (blue). (Scale bars, 50 μm.)

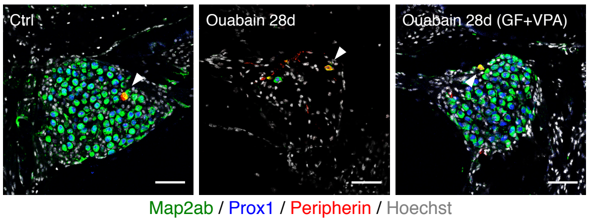

Supplemental Figure 3. Type I and Type II SGNs before and after injury in the SG.

Left: Prox1-positive Type I and Peripherin-positive Type II SGNs in intact SGNs. Middle: Type I but not Type II SGNs dramatically decreased 28 days after ouabain administration. Right: Type I but not Type II SGCs were regenerated when treated with a combination of GFs and VPA after ouabain administration. The arrows indicate Peripherin-positive Type II SGNs. (Scale bars, 50 μm.)
